# Selections and Abstracts

**Published:** 1908-05

**Authors:** 


### SELECTIONS AND ABSTRACTS

### DOCTOR AND DRIVER.

The *British Medical Journal* relates the following story: Dr. ———, of Baltimore, was awakened one stormy night recently by a man who declared the doctor's services were wanted three miles out in the country. Just before the doctor called up the stable for his horse, the visitor asked what the charge would be. "Three dollars," was the reply. When the house containing the supposed patient was reached the man alighted first, and handing the doctor three dollars, remarked: "You needn't come in doctor. You see it is this way: No hackman would drive me out for less than six dollars, and it occurred to me that your horse might need exercise." The tale has more than one bitter moral. The doctor is expected to be at the beck and call of every one at any hour of the day or night; he is also expected to accept a fee which an ordinary workman would decline without thanks; and the worst of it is, the doctor so often does what is expected of him.—*Transvaal.*

### NEW LAW FOR GERMANY.

### SUPPLEMENTARY MEDICAL PRACTICE ACT PROPOSED.

The German medical journals contain the text of a bill now before the Reichstag which provides for the regulation of the practice of medicine by irregulars. This bill, which has caused considerable editorial comment, provides for the regulation of a large class of irregular practitioners, who are patronized by the public, but who are not qualified as physicians. The editorials admit that the bill is not perfect, but urge the medical profession to work for its enactment, as it is a step in the right direction and a gain over present conditions, especially so far as public health is concerned.

The bill provides that every person who makes a practice of treating disease or morbid conditions of any kind, and who is not a legally qualified physician, must register his name and address with the police, record removals from time to time and keep a set of books open to the inspection of the police at all times. Persons known to have been convicted of crime or those whose methods are liable to injure the health of man or animals are not to be allowed to register or practice. Persons registering under this law are not to be allowed to treat venereal diseases. Public advertisement for remedies for such diseases as well as advertisement of aphrodisiac remedies for "loss of vigor," measurees to prevent conception or to interrupt pregnancy, is entirely prohibited. The exploitation of any remedy liable to injure the health or to defraud the purchaser can be prohibited by a committee of the bundesrath. Advertisement of remedies, the composition of which or the essential factors in the manufacture of which are secret, is also forbidden. Practitioners registering under this law are forbidden to use any treatment not based on personal examination. This will preclude the use of "absent treatment." They are not allowed to use any narcotics except local anesthetics, neither are they permitted to use hypnosis or "mystic processes" in their treatment.

A commission formed by the Imperial Health Office is to be established by the bundesrath for the purpose of passing on remedies or substances claimed to be serviceable for the treatment of diseases of man or animals. This commission is to consist of a judge and of experts from the professions of medicine, veterinary science and pharmacy. Appointment is for five years. Making claims by advertisements or otherwise of properties possessed by remedies not borne out by the findings of the commission is punishable by imprisonment, fine, or both. The same penalty is to be applied to those making knowingly untrue statements with reference to the person of the manufacturer or originator, or to the person or persons responsible for the publication of the advertisement. Fine and imprisonment are also imposed on proof of the following: Soliciting for absent treatment by public announcement or advertisements; publicly announcing remedies for the prevention or cure of venereal diseases; publicly announcing or recommending remedies for the treatment of disease of animals or man in which the constituents or essential character of the cure, or the process of preparation is kept secret or is obscured.

The *Munchener medizinische Wochenschrift* says editorially: "The bill does not in any sense fulfil the wishes of those physicians who seek the abolition of quackery. It contains, however, a number of practical measures for the restriction of quackery so that the removal of the worst outgrowths of this injury to the public health may be hoped for. Registration and the necessity of keeping books open to the inspection of the police will be a decided hindrance to the business of the quack. The other provisions of the bill contain much that is good. It should be the business of the physician to recognize in the bill what is positively offers rather than to complain about what is lacking, since a vigorous opposition is to be expected from those interested in the defeat of the bill."

The measure outlined above is of importance, since it introduces a new principle in medical legislation, namely, the regulation of irregulars, quacks and fakers by the local police rather than by the state. These people are granted the right to practice under police surveilance. All efforts made in this country so far have been for the prevention of the practice of medicine by anyone except those legally qualified to do so. When a sect or cult became so powerful that it could not be prevented from practicing, the difficulty has been met by admitting representatives of the sect and according them to the legal standing of physicians, regardless of their scientific standing. Recent legislation along osteopathic lines in a number of states is an example. The proposed German law attempts to meet the difficulty by allowing the followers of those fads and cults which a portion of the public wish to patronize to carry on their business, but under police surveilance and without at the same time lowering the standing and personnel of the recognized medical profession. This plan is worthy of careful study. The progress of the bill and the practical results of the law, if adopted, will be watched with interest.—*Journal Medical Economics, April* 11, 1908.

